# Leproma's dermoscopy^☆^^☆☆^

**DOI:** 10.1016/j.abd.2019.09.027

**Published:** 2020-03-20

**Authors:** Anna Carolina Miola, Natalia Parenti Bicudo, Giuliane Minami Tsutsui, Helio Amante Miot

**Affiliations:** a Department of Dermatology and Radiotherapy, Universidade Estadual do Estado de São Paulo, Botucatu, SP, Brazil; b Department of Dermatology, Instituto Lauro de Souza Lima, Bauru, SP, Brazil

The prevalence of leprosy has been declining steadily throughout the world since the 1980s as a result of multidrug therapy.^1^, ^2^ In Brazil, it is still endemic in many regions, and delayed diagnosis, especially in multibacillary forms, is one of the main factors in the maintenance of contagion.

Characteristically, leprosy evolves in a chronic, indolent form, with polymorphous lesions and in an oligosymptomatic form. Virchowian forms are even more indolent and diffuse infiltration can make diagnosis difficult. Lepromas, although asymptomatic, are often the reason for seeking medical care, and may generate diagnostic confusion with other papulonodular dermatoses such as granuloma annulare, dermatofibroma and sarcoidosis, among others.

As Virchowians are the most bacilliferous, all strategies for identification and early treatments are valuable. In this manuscript, the dermoscopic findings of the lepromas are described.

Male, 54 years old, brown, with asymptomatic nodular lesions throughout the integument one year ago. At examination, multiple normochromic papules of fibromatous consistency were found, associated with diffuse infiltration of the skin and diffuse loss of hair on the body (Fig. 1). These nodules, at dermoscopy, had diffuse yellowish coloration, with a discrete brownish halo and, at the center of the lesion, multiple heteromorphic telangiectasias more concentrated in the periphery and cicatricial nacreous aspect at the center of the lesion (Figure 2, Figure 3, Figure 4). Patient reported family cases and had treated leprosy for about 20 years, but was unaware of the schedule or treatment time used at the time. At anatomopathological examination, epidermal rectification associated with diffuse infiltration of xanthomatous macrophages was observed in the superficial and deep dermis. Fite-Faraco staining revealed multiple globules of viable bacilli within these macrophages, and earlobe and leproma baciloscopy result in a bacilloscopic index of 6+ with innumerable intact bacilli (Fig. 5).

**Figure 1:**
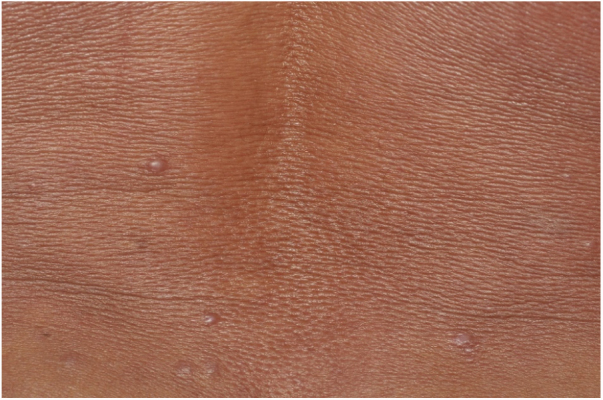
Presence of multiple normochromic nodules associated with diffuse cutaneous infiltration of the dorsum.

**Figure 2:**
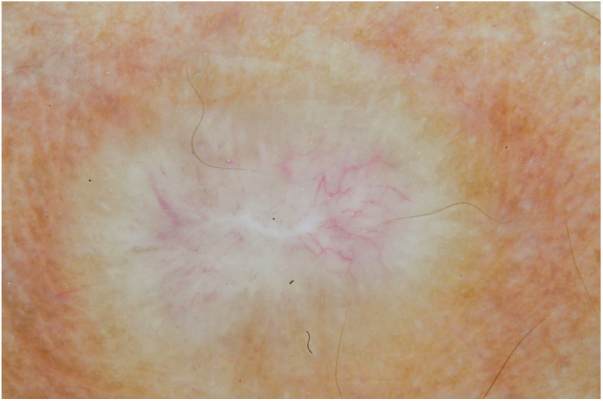
Dermoscopy of the leproma: a yellowish appearance, with a scarring nacreous center and telangiectasias of a centrifugal character. (Contact technique/alcohol immersion).

**Figure 3:**
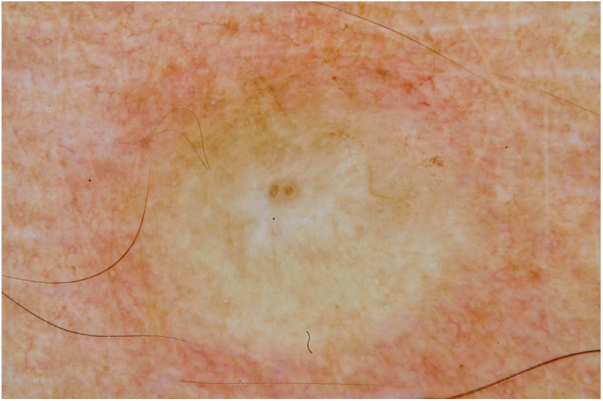
Dermoscopy of the leproma: presence of light brown halo in the lesion, with a yellowish appearance, with a scarring nacreous center and telangiectasias of a centrifugal character. (Contact technique/alcohol immersion).

**Figure 4:**
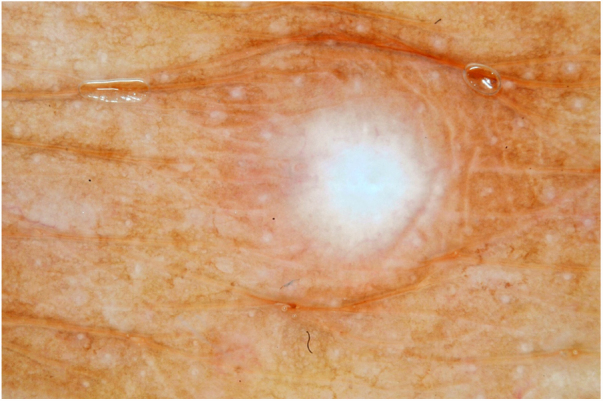
Leproma's dermoscopy: scarring nacreous area at the center of the lesion.

**Figure 5:**
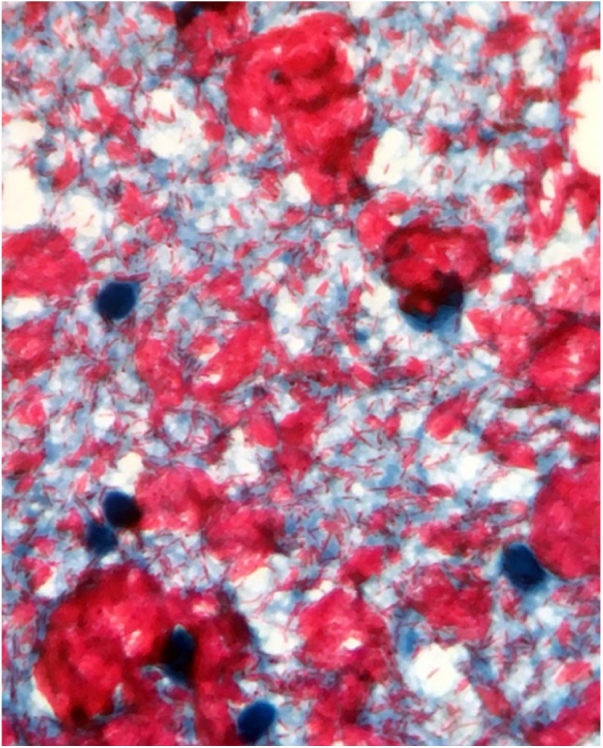
Baciloscopy of earlobe and leproma: bacilloscopic index of 6+ with innumerable bacilli intact.

Individuals that evolve to the virchowian pole, such as this case, present humoral immune response, with high production of antibodies against the PGL-1 antigen, but these antibodies are ineffective in the elimination of *M. leprae*, with consequent multiplication and bacillary spread through the haematogenic pathway.^2^, ^3^ In virchowian patients, skin lesions tend to be multiple and symmetrical, localized, poorly delimited, associated or not with generalized cutaneous infiltration, which can progress to infiltrated papules, plaques and nodules, called lepromas, which may be arranged throughout the integument, in few or large number of lesions.^1^

Dermoscopy, in addition to being of great importance in the diagnosis of cutaneous tumors, is currently used in the detection and diagnosis of other non-tumoral skin diseases, such as inflammatory and infectious dermatoses, in order to reinforce clinical suspicion and differential diagnosis.^4^ Currently, there are, in the literature, dermatoscopic descriptions of numerous dermatoses, such as psoriasis, lichen planus, sarcoidosis, annular granuloma, dermatofibroma, among others.

The dermoscopic description of the lesions of some clinical forms of leprosy can be found in the literature, such as the histoid and tuberculoid forms.^5^, ^6^ To date, there are no descriptions of the dermoscopic features of lepromas. Its dermatoscopic appearance is similar to the nodules described in the histoid form of leprosy, with fine telangiectasias associated with a halo of brownish pigmentation, despite the anatomopathological differences between the two lesions, since a lesion of leprosy in the histoid form presents multiple fusiform histiocytes grouped in strip, different from the xanthomatous macrophages commonly found in lepromas.^7^

Its yellowing color in the dermoscopic examination is due to the intense xanthomization of the lepromes (Virchow cells), which is less evident in other lesions such as granuloma annular, sarcoidosis and xanthogranuloma,^8^, ^9^ which show orange color due to the fusiform appearance of the macrophages that form them. Already the peripheral brownish pigmentation of the lesion in dermoscopy may be more difficult to observe in patients with a higher phototype.

In anatomopathological examination of the leproma, there is an epidermal rectification, probably due to the accumulation of macrophages grouped in the superficial and deep dermis, with clear cytoplasm and vacuolated aspect. These vacuoles, in the coloration of fite-Faraco, contain a large quantity of bacilli; forming globes.^7^ Vascular ectasias in the superficial dermis may also be visualized. In view of the dermoscopic observations, the correlation of the visualized structures with the alterations in the examination can be performed, so that the presence of numerous macrophages containing globules confer the nodular appearance of the lesion, and the vascular ectasias are represented by the thin telangiectasias seen on examination. Such dermatoscopic features make a differential diagnosis with dermatoses such as sarcoidosis and lupus vulgaris, among others.^5^, ^6^, ^7^, ^8^ Its is important to note that, in the case described, only contact dermatoscopy was performed; dermatoscopy with polarized light without contact could bring additional details in the description of the lesion.

The diagnosis of leprosy, however, is clinical, so that dermatoscopic aspects, as well as laboratory tests, anatomopathological and specific staining help in cases of greater clinical difficulty. Treatment with the diagnosis should be done promptly, as well as investigation of communicators, in order to reduce the transmission of the bacillus and controlling the incidence of new cases of the disease in the country.^10^

## Financial support

None declared.

## Authors’ contributions

Anna Carolina Miola: Approval of the final version of the manuscript; elaboration and writing of the manuscript; obtaining, analysis, and interpretation of the data; critical review of the literature; critical review of the manuscript.

Natalia Parenti Bicudo: Approval of the final version of the manuscript; obtaining, analysis, and interpretation of the data; critical review of the manuscript.

Giuliane Minami Tsutsui: Approval of the final version of the manuscript; intellectual participation in the propaedeutic and/or therapeutic conduct of the studied cases, critical review of the manuscript.

Helio Amante Miot: Approval of the final version of the manuscript; elaboration and writing of the manuscript; critical review of the literature; critical review of the manuscript.

## Conflicts of interest

None declared.
